# Supplementary material for: A cloud-assisted key agreement protocol for the E-healthcare system
Source: PLoS One. 2025 Jun 3;20(6):e0322313. doi: 10.1371/journal.pone.0322313 (PMC12133001; doi:10.1371/journal.pone.0322313)
Supplement: Attachment — Submitted filename: Response to Reviewers.docx [file pone.0322313.s003.docx]

To,

The Editor in Chief

PLoS ONE

Subject:	***REBUTTAL LETTER***

I am submitting a revised, modified version of the manuscript entitled “A Cloud-Assisted Key Agreement Protocol for the E-Healthcare System” for onward submission to the panel of reviewers for consideration and possible acceptance. The table mentioned below demonstrates the point-by-point response to reviewers’ concerns. Please let me know of your decision as soon as possible.

| **JOURNAL REQUIREMENT** | | | |
| --- | --- | --- | --- |
| **Comment#** | **Description** | | **Author Response** |
| 01 | Please ensure that your manuscript meets PLOS ONE's style requirements, including those for file naming. The PLOS ONE style templates can be found at  <https://journals.plos.org/plosone/s/file?id=wjVg/PLOSOne_formatting_sample_main_body.pdf>  and  [https://journals.plos.org/plosone/s/file?id=ba62/PLOSOne_formatting_sample_title_authors_affiliations.pdf](https://track.editorialmanager.com/CL0/https:%2F%2Fjournals.plos.org%2Fplosone%2Fs%2Ffile%3Fid=ba62%2FPLOSOne_formatting_sample_title_authors_affiliations.pdf/1/010f0194fb48c171-f86e05dc-c9ec-43d8-90d7-c06d9e807ea6-000000/aWn3G9DXfD1VrOBhb0vzOXRwzpK8VPcPcSPvZTgmHNQ=197) | | Thanks, the prescribed format has been followed |
| 02 | We note that the grant information you provided in the ‘Funding Information’ and ‘Financial Disclosure’ sections do not match.  When you resubmit, please ensure that you provide the correct grant numbers for the awards you received for your study in the ‘Funding Information’ section. | | This research doesn’t receive any funding. |
| **REVIEWER#01** | | | |
| **Comment#** | **Description** | | **Author Response** |
| 01 | The abstract did not clarify the criteria that the researcher adopted to show that the proposed method is better than others, nor the numerical values of the most important of these criteria. | | Thanks, the abstract is revised  Secure data transmission is critical to cloud-based electronic healthcare (e-healthcare) systems. Upon delving into the literature, it becomes clear that several security mechanisms have been developed to ensure the security of exchanged information across patients, physicians and the cloud server, but they do not resist vulnerabilities such as man-in-the-middle, side-channel, and session key hijacking attacks. These vulnerabilities can seriously compromise the integrity of exchanged medical records. In light of this, the article proposes a cloud-assisted key agreement protocol for the e-healthcare system to enable secure authentication for patient monitoring, enhancing mutual authentication of the participating entities and creating secure session keys for secure open-channel communication. The proposed methodology employs robust and lightweight procedures, including SHA-256 and Elliptic Curve Cryptography (ECC), while considering the challenges of implementing strong security protocols in healthcare systems, such as the need for high performance and low energy consumption. The proof of correctness and robustness of the proposed protocol has been analyzed through the Real-Or-Random (RoR) model, ProVerif verification toolkit and pragmatic illustration, while the efficiency and efficacy were checked by measuring computation, communication, storage costs, and energy consumption. The result obtained from this analysis demonstrated that the proposed protocol is 46.99% better in communication, 96.46% in computation, and 53.69% in energy consumption, which is installing its superiority over its competitors. Hence, it is recommended for practical implementation in the real-world cloud-based e-healthcare scenario. |
| 02 | The keywords are general and do not contain any specification. | | Thanks, replaced with newer keywords  **Keywords:** Cloud Computing, Pi-Calculus, Protocol, Authentication, Encryption, Patient Monitoring, ECC |
| 03 | The researcher's contributions are very general and the distinctive contribution made by the researcher in this manuscript is not specified. It is possible to include the contributions as a paragraph in the introduction. In addition, the introduction does not contain a paragraph explaining the structure of the research in general. | | Now, revised as per guidelines  To propose a key-agreement protocol for a cloud-assisted e-healthcare system operating without passwords or user biometrics that has the potential to significantly improve security by eliminating online/offline password guessing, password breaches, and compromised biometric data using elliptic curve cryptography (ECC) and SHA256 methods.  To comprehensively analyze the proof of correctness and robustness of the proposed key agreement protocol employing the well-known and widely used Real-Or-Random (ROR) model and ProVerif simulation, confirming integrity, secrecy and confidentiality of patient-sensitive information.   - To methodologically tesbed the performance metrics of the proposed key agreement scheme through a testbed research method, confirming its effectiveness, strength, and efficacy.   To analyze the proposed protocol for performance metrics comparatively and check how well it balances with security, the often conflicting aspects of security and performance are frequently ignored in previous studies. |
| 04 | The research paper lacks much clarification through figures or diagrams, in addition to the lack of a discussion that clarifies the researcher’s reading of the results he obtained and comparing them with other previous works. | | More Figures are added |
| 05 | The conclusions are not based on numerical values of the results and the proposed future work is not clearly explained. | | Revised |
| **REVIEWER#02** | | | |
| **Comment#** | **Description** | **Author Response** | |
| 01 | Towards the end of the abstract, give a summary of the key findings of this study. This may include the security threats prevented by your proposed scheme as well as the computation costs, communication costs, storage overheads, and energy consumption. | Thanks,  The result obtained from this analysis demonstrated that the proposed protocol is 46.99% better in communication, 96.46% in computation, and 53.69% in energy consumption, which is installing its superiority over its competitors. Hence, it is recommended for practical implementation in the real-world cloud-based e-healthcare scenario. | |
| 02 | The quality of English used in this paper must be greatly enhanced. | Enhanced | |
| 03 | Rewrite the introduction section so that the problem domain in vividly clear to the readers. | Yes, Thanks  The whole introduction is revised form scratch | |
| 04 | The motivation section is not well written since you have failed to describe the shortcomings of the current mutual authentication and key agreement protocols. | This concern is also addressed by re-writing the motivation  Strict authentication of all the participating entities must be followed because the cloud-based e-healthcare infrastructure designated for patient health information is extremely sensitive and needs to be protected against abuse, breaches, and unauthorized access. The data sent between sensors, wearables, and cloud servers is susceptible to numerous threats, diversification, and interruption without secure key agreement methods. Also, security is an urgent necessity for Internet-connected devices to protect data and the devices themselves from potential breaches. Healthcare professionals, IT security experts, and e-healthcare system developers play a crucial role in ensuring the security of these systems. Unfortunately, many IoT devices have inadequate security measures to address contemporary threats, and e-healthcare providers occasionally mismanage their security features. Introducing network capabilities to embedded devices, wearables, or IoT requires the development of a secure authentication protocol, which is a challenging task that demands careful consideration and strategic planning, and it is essential to design a security protocol to secure the whole system and stakeholders. Therefore, the need to address critical security, privacy, and efficiency issues in exchanging and storing sensitive patient data drives the design of a cloud-assisted key agreement protocol in the e-healthcare system. A key agreement protocol guarantees that only authorized parties (such as patients, physicians, and healthcare professionals) may access the data by creating a secure cryptographic method for key generation, encryption and decryption of the exchange of sensitive information among the participating entities. | |
| 05 | Your contributions do not reflect any novelty: ECC and SHA-256 are well-known cryptographic techniques. What is so unique about your proposed scheme that differentiates it from previous ECC-based and SHA-256 based schemes? | Correction has been made | |
| 06 | Your third contribution reads as follows: "The proposed protocol's performance is tested by measuring computation, communication, strong overheads, and energy consumption, ensuring its practicality and reliability for real-world implementation."  ---Replace 'strong overheads' with 'storage overheads'. Proof-read the entire paper to eliminate similar grammatical and spelling mistakes--- | Thanks for pointing this technical concern,  The correction has been made | |
| 07 | Before introducing the Related works, add a paragraph to describe how this paper is organized. | Thanks, the following paragraph has been added  The remainder of the article is organized as follows: in section 2, the review of existing schemes was discussed, and their pros and cons are presented in the form of a table; in section 3, the detailed system architecture was given in which entities' roles, how it will work and associated aspects were given in textual as well as diagrammatically; in section 4, the proposed key agreement scheme is presented in three phases, including setup, registration, and authentication; the algorithmic representation has been given for the proposed scenario, emphasizing its practical implications. Section 5 demonstrates the security analysis, both formally using the RoR model and informally through pragmatic discussion; section 6 considers the performance and comparative analysis, while section 7 argues what has been done in this article for the easy understandability of the readers. | |
| 08 | In the related works section, do the following: | Thanks, the whole section is modified | |
|  | i ) Describe the techniques used in the related works, as well as the pros and cons of all presented works |  |  |
|  | ii) Add a table to summarize the presented works in terms of the techniques deployed, pros and cons |  |  |
|  | iii) After the table in (ii) above, add a paragraph to summarize all the identified research gaps. Thereafter, describe how your proposed solution helps alleviate these challenges. |  |  |
| 09 | For enhanced readability, the various phases of your proposed protocol must be presented in a step-wise manner. | Thanks, the whole section is revised | |
| 10 | Under the 'Proposed Protocol' section, you have claimed that the proposed scheme included setup, registration, and key agreement phases. However, the abstract indicated that you are developing a cloud assisted key agreement protocol for the e-healthcare system to enable secure authentication for patient monitoring, enhancing mutual authentication of the participating entities and creating secure session key among all them for secure open channel communication.  --- Where is the mutual authentication aspect of your protocol? Is it not supposed to be one of the key phases in your proposed protocol---? | Key agreement phase basically mutually authenticate all the participating entities, therefore, keeping in view your concern the whole section has been modified, and the concern is addressed up to a maximum extent. | |
| 11 | Use proper names to name your algorithms and figures. You have used Module 1 and Module 2, which are quite confusing. | Thanks, correction has been made by renaming the algorithm | |
| 12 | Under 'ProVerif Simulation', you claim that This toolkit simulated the launch of a man-in-the-middle attack and found that the proposed protocol meets the needed security features, such as secrecy, authentication, and process equivalencies.  -- How can readers verify the claim you make above from the ProVerif output---?  --You need to describe the output of the ProVerif output--- | Yes, after the summary of the result, the point is explained for the readers.  The code provided includes the definition of two queries, which allows for a thorough examination and the identification of potential loopholes or the simulation of malicious user actions. The above mentioned result summary from this code, a key component of our assessment, demonstrates that the SK remains uncompromised, and an attacker would be unable to exploit it from an open line. This leads to the conclusion that a man-in-the-middle attack, insider attack, forgery attack, and session key hijacking attack are all infeasible on the proposed protocol. | |
| 13 | Under the 'Pragmatic Discussion', add more attack vectors | Thanks, more discussion regarding different attacks have been made | |
| 14 | In your third contribution, you claimed to have analyzed your proposed protocol in terms of energy consumption. However, this has not been presented under the 'Performance Analysis' section. You must present this energy consumption analysis. | Yes, the proposed protocol is also analyzed for energy consumption.  The resources use a certain amount of battery power while the proposed key agreement protocol is executed as soon as its initialization phase is run. The amount of power consumed by the Raspberry Pi, laptop, and cell phone for calculating the session secret key is represented by the equation E_X_=C_Y_ x C_Z_ for a wireless channel [51]. The reliability of the proposed key agreement protocol is evident in its consistent power consumption. Suppose the computation costs of the proposed key agreement protocol is C_Y_, which is 87.44 ms, and suppose C_Z_ is the CPU's maximum power consumption, which is fixed and equal to 10.88 Watts, according to [52-53] for wireless data transmission. By entering these numbers into the calculation, E_X_ = 87.44 x 10.88 = 874.4 mJ or 0.88 joule. Therefore, 0.88-joule power is consumed upon running the proposed key agreement protocol for security authentication all the participating entities in the cloud computing paradigm and computes session secret key SK. | |
| 15 | The greatest weakness of the 'Performance Analysis' section is that no comparative evaluation has been carried out. You must compare the performance of your protocol against the state-of-the-art protocols in this domain. In addition, you must describe and interpret all the obtained results. | Yes, the proposed protocol is also analyzed comparatively with state of the art work  When comparing the proposed protocol with Mohit et al. [47], Li et al. [48], Sahoo et al. [49], and Zhou et al. [50] in terms of performance metrics (communication and computation costs), the result in Table 7 provides a comprehensive comparison. It demonstrates that the proposed key agreement protocol is lightweight and robust, as shown diagrammatically in Fig 5, and outperforms the existing protocols in these aspects.  The proposed protocol stands out with its exceptional performance. It is 41.57% better in terms of communication cost than Mohit et al. [47], 24.22% from Li et al. [48], 28.35% from Sahoo et al. [49], and 46.99% from Zhou et al. [50]. Similarly, it is 58.08% better in terms of computation costs from Mohit et al. [47], 96.46% from Li et al. [48], 9.35% from Sahoo et al. [49], and 21.47% from Zhou et al. [50]. These results underscore the overall outstanding performance of the proposed protocol. | |
| 16 | The conclusion section is poorly done and it fails to capture the key findings, practical implications of the obtained results, limitations of your proposed protocol, as well as future research directions. | The conclusion is revised  This research introduces a cloud-assisted key agreement protocol designed for secure communication within the e-healthcare system, utilizing ECC and SHA256 cryptographic primitives. The proposed method facilitates cross-verification among the parties involved and ensures the legality of participating entities in the cloud computing paradigm. What sets this protocol apart is the thoroughness of its examination, which includes formal security proofs utilizing the RoR model and ProVerif simulation, as well as informal security proofs through pragmatic illustrations. This comprehensive approach has been evaluated by considering computation, communication, storage costs, and energy consumption metrics, with results indicating that it is lightweight, effective for cloud setting, robust against known threats, and fulfills essential security requirements. Comparisons with state-of-the-art schemes reveal that the proposed protocol outperforms others regarding communication and computation costs, thus affirming its applicability in the real-world scenario. In future, the same e-healthcare setting can be secured by designing a protocol using advanced AI and machine learning algorithms and can be explained with the help of a real-world use case. | |

**DR. ISMAIL KESHTA**

**College of Applied Sciences**

**Department of Computer Science and Information Systems**

**AlMaarefa University**

**Riyadh, Saudi Arabia**

**e-mail:** [imohamed@um.edu.sa](mailto:imohamed@um.edu.sa)
