# Supplementary material for: A cloud-assisted key agreement protocol for the E-healthcare system
Source: PLoS One. 2025 Jun 3;20(6):e0322313. doi: 10.1371/journal.pone.0322313 (PMC12133001; doi:10.1371/journal.pone.0322313)
Supplement: Attachment — Submitted filename: Response_to_Reviewers_auresp_2.docx [file pone.0322313.s004.docx]

To,

The Editor in Chief

PLoS ONE

Subject:	***REBUTTAL LETTER (2^nd^ Round Submission)***

I am submitting a revised, modified version of the manuscript entitled “***A Cloud-Assisted Key Agreement Protocol for the E-Healthcare System***” for onward submission to the panel of reviewers for consideration and possible acceptance. The table mentioned below demonstrates the point-by-point response to reviewers’ concerns. Please let me know of your decision as soon as possible.

| **REVIEWER#02** | |
| --- | --- |
| **Comment/Concern** | **Author’s Response and Action** |
| 1) The quality of English used in this paper is still low. For instance:  "Lopes et al. [12] proposed a protocol to provide safe and reciprocal device authentication within the system, vulnerable to traceability attacks."  ---but is vulnerable to traceability attacks.  "and 53.69% in energy consumption, which is installing its superiority over its competitors."  --'installing' is not appropriate in the above sentence. | Yes, the correction has been made,  ~~Installing~~ changed to inaugurating |
| 2) In the abstract, you have still failed to state some of the security threats prevented by your proposed scheme. | Great point, thanks a lot  The highlighted concern has been addressed, as shown in as under:  The result obtained from the security analysis demonstrated that the proposed protocol resisting man-in-the-middle, replay, DoS, traceability/tracking, desynchronization, impersonation, and side channel attacks offers key secrecy, confidentiality, integrity, and authorization. In contrast, the result depicted from the performance analysis section shows that the proposed protocol is 46.99% better in communication, 96.46% in computation, and 53.69% in energy consumption, which is inaugurating its superiority over its competitors. Hence, it is recommended for practical implementation in the real-world cloud-based e-healthcare scenario. |
| 3) In the motivation section, describe some of the shortcomings of the current mutual authentication and key agreement protocols. | Thanks for pointing out such a critical point, the motivation is revised  The existing authentication protocols presented by different researchers from time to time (discussed in detail in the related work section of this article) for cloud-assisted e-healthcare systems either suffered from privacy, anonymity, efficiency, and design issues or failed to resist impersonation, DoS, insider, desynchronization, replay, MITM, and side-channel attacks. Some of these protocols suffer from scalability issues, complexity in implementation, and limited device compatibility challenges, while others are susceptible to specific types of attacks, such as ESL, tracking/traceability attacks, or session key hijacking issues, and some of them have maximum computation due to modular exponentiation.  Furthermore, a robust authentication of all the participating entities must be followed because the cloud-based e-healthcare infrastructure designated for patient health information is extremely sensitive and needs to be protected against abuse, breaches, and unauthorized access. The data sent between sensors, wearables, and IoT devices to the cloud server is susceptible to numerous threats, modifications, and interruptions by adversaries without secure key agreement methods. The inadequacy of current security measures is a pressing issue that demands improvement and innovation. Healthcare professionals, IT security experts, and developers play a crucial role in ensuring the security of said cloud-assisted e-healthcare system, but unfortunately, internet-connected devices (sensors, wearables, or IoT devices) are resource-constrained and lack inadequate security measures to address contemporary threats, and the said service providers occasionally mismanage their security features. It is also necessary to introduce network capabilities in these network-enabled devices, which is needed for a dynamic authentication protocol (like the protocol proposed in this article), but it is too challenging and needs careful consideration and strategic planning, which is often lacking in current cloud service providers. Considering all the aforementioned issues and challenges, there is a need to address the critical security, privacy, and efficiency problems in the exchanged patient-sensitive information by designing a cloud-assisted key agreement protocol in the e-healthcare system that can guarantee to mitigate all the weaknesses discussed above. |
| 4) For each of your research contributions, vividly bring out the uniqueness/novelty compared with other related state-of-the-art solutions. | Thanks --- Revised  The contributions of this research are summarized in bullet points, highlighting the innovative application of advanced technologies in developing this new protocol.   - To propose a key-agreement protocol for a cloud-assisted e-healthcare system operating without passwords or user biometrics that has the potential to significantly improve security by eliminating online/offline password guessing, password breaches, and compromised biometric data by utilizing a lightweight and robust cryptographic technique called elliptic curve cryptography (ECC) and a collision-free one-way hash cryptographic function called SHA256 algorithm. - To comprehensively analyze the proof of correctness and robustness of the proposed key agreement protocol employing the well-known and widely used Real-Or-Random (ROR) model and ProVerif simulation, confirming integrity, secrecy, confidentiality, and reachability of the patient-sensitive information exchanged in a potentially hostile environment. - To methodologically evaluate the performance metrics of the proposed key agreement scheme using a testbed research method, confirming its effectiveness, strength, and efficacy while considering computation, communication, storage costs, and energy consumption. - To comparatively analyze the proposed protocol for performance metrics in checking how well it balances with security, the often conflicting aspects of a protocol are frequently ignored in previous studies. |
| 5) In the Related works section, you have attempted to point out the weaknesses of the current works. However, you have not provided the rationale for your critique. For instance, what makes you believe that the scheme in [12] is vulnerable to traceability attacks? Why do you think that the protocol in [13] has design issues? --You must either cite or give rationale for the critique provided. This increases the credibility to the identified research gaps in the related works. | Dear Sir, The proof you are demanding occupies too much space. For instance, I am analyzing once again to confirm it, but I assure you that after conducting a thorough investigation, I have identified and critiqued the loopholes and mentioned them. It is essential to note that only individuals with the proper knowledge can mention the criticism of someone's work.  The whole section is revised |
| 6) Replace 'Key Agreement Phase' with 'Authentication and Key Agreement Phase | Revised as suggested |
| 7) My previous COMMENT # 11 was as follows:  "Use proper names to name your algorithms and figures. You have used Module 1 and Module 2, which are quite confusing."  --You have indicated that you have made corrections above. However, in Registration section, you have still written as follows:  >>The system ensures the security of the communication by sending {s, X_P_, H_P_, K_P_} back to the patient terminal, where it is stored in its memory, as shown in module 1."  >> Module 1. Patient Registration  >>This data is then stored in the cloud server's memory, as shown in module 2, ensuring the highest level of security.  >>Module 2. Physician Registration  --There are so many instances where you still use 'Module' instead of appropriate names such as 'Figure'. Ensure that all changes you effect are reflected in the entire paper. | Thanks for highlighting such a technical mistake, Great  I have changed all the module into figures, now, the current figures order in the article are as under:  **S1 Fig 1. System Architecture.** (PDF)  **S1 Fig 2.** Sensor/Wearable Registration**.** (PDF)  **S1 Fig 3.** Mobile Device Registration**.** (PDF)  **S1 Fig 4.** Authentication and Key Agreement**.** (PDF)  **S1 Fig 5.** Algorithmic representation of the proposed protocol**.** (PDF)  **S1 Fig 6.** ProVerif Validation – Summary of Verification**.** (PDF)  **S1 Fig 7. Computation Cost of the Proposed Protocol.** (PDF)  **S1 Fig 8. Communication Cost of the Proposed Protocol.** (PDF)  **S1 Fig 9. Storage Cost of the Proposed Protocol.** (PDF)  **S1 Fig 10. Comparative Analysis – A diagrammatic overview.** (PDF)  While the one file is TXT which is simulation code of ProVerif software verification toolkit  **S2 File 1. ProVerif Simulation Code.** (TXT) |
| 8) In Table 7, you have provided some comparative analysis in terms of computation and communication costs. However, you have failed to include storage costs and energy consumption in this Table 7. | Thanks a lot  Comparative Analysis in terms of energy consumption has been added  Storage cost has not been found in the mentioned schemes – therefore avoided |

**DR. ISMAIL KESHTA**

**College of Applied Sciences**

**Department of Computer Science and Information Systems**

**AlMaarefa University**

**Riyadh, Saudi Arabia**

**e-mail:** [imohamed@um.edu.sa](mailto:imohamed@um.edu.sa)
